# Predicting Missing Marker Trajectories in Human Motion Data Using Marker Intercorrelations

**DOI:** 10.1371/journal.pone.0152616

**Published:** 2016-03-31

**Authors:** Øyvind Gløersen, Peter Federolf

**Affiliations:** 1 Department of Physics, University of Oslo, Oslo, Norway; 2 Department of Physical Performance, Norwegian School of Sport Sciences, Oslo, Norway; 3 Department of Neuroscience, Faculty of Medicine, Norwegian University of Science and Technology, Trondheim, Norway; 4 Department of Sport Science, University of Innsbruck, Innsbruck, Austria; J. Heyrovsky Institute of Physical Chemistry, CZECH REPUBLIC

## Abstract

Missing information in motion capture data caused by occlusion or detachment of markers is a common problem that is difficult to avoid entirely. The aim of this study was to develop and test an algorithm for reconstruction of corrupted marker trajectories in datasets representing human gait. The reconstruction was facilitated using information of marker inter-correlations obtained from a principal component analysis, combined with a novel weighting procedure. The method was completely data-driven, and did not require any training data. We tested the algorithm on datasets with movement patterns that can be considered both well suited (healthy subject walking on a treadmill) and less suited (transitioning from walking to running and the gait of a subject with cerebral palsy) to reconstruct. Specifically, we created 50 copies of each dataset, and corrupted them with gaps in multiple markers at random temporal and spatial positions. Reconstruction errors, quantified by the average Euclidian distance between predicted and measured marker positions, was ≤ 3 mm for the well suited dataset, even when there were gaps in up to 70% of all time frames. For the less suited datasets, median reconstruction errors were in the range 5–6 mm. However, a few reconstructions had substantially larger errors (up to 29 mm). Our results suggest that the proposed algorithm is a viable alternative both to conventional gap-filling algorithms and state-of-the-art reconstruction algorithms developed for motion capture systems. The strengths of the proposed algorithm are that it can fill gaps anywhere in the dataset, and that the gaps can be considerably longer than when using conventional interpolation techniques. Limitations are that it does not enforce musculoskeletal constraints, and that the reconstruction accuracy declines if applied to datasets with less predictable movement patterns.

## Introduction

Loss of marker-information due to, for example, occlusion or marker detachment [1] often imposes challenges in marker based motion analysis [2]. Currently, the standard methods for filling gaps in marker trajectories are linear or spline interpolation, or reconstructing the trajectory in a local coordinate frame [3]. However, these approaches are restricted to gaps of short duration or to rigid body segments carrying 4 or more markers.

Several additional approaches for the “missing marker problem” have been proposed [4–10]. These methods utilize the high covariance between marker coordinates that is typical for human motion tracking data [11, 12] and reconstruct missing markers from the information provided by the available markers. A proof-of-principle analysis of an approach utilizing principal component analysis (PCA) showed promising results for relatively long datasets (20 stride cycles) with gaps in a single marker [4]. However, to be generally applicable, the method needs to be extended for data with gaps in multiple markers. Also, the method’s capability to successfully reconstruct datasets with less repetitive or predictive movements, for instance during a gait transition phase, needs investigation.

The purpose of the current study was therefore to further develop the strategy proposed in [4] for situations with multiple gaps, and to test this algorithm on datasets containing less predictable human movements. Specifically, the movements tested were the transition from walking to running, the gait of a child with Cerebral palsy, and the movement pattern of a healthy person walking on a treadmill.

## Methods

The underlying idea of the reconstruction algorithm is to project the incomplete marker data into a basis where it is sparsely represented, apply a coordinate transformation, and then transform it back into the original coordinates to obtain an estimate of the missing marker’s coordinates. The following section summarizes the conceptual outline presented in [4], and describes the modifications proposed in the current study.

### PCA-based reconstruction of a single missing marker

We consider the situation where we have captured the kinematics of a human posture represented by *m* markers at *n* discrete time points, and that these measurements are conjoined into a n×3m-measurement matrix ***M***. For now we assume that one and only one marker trajectory in ***M*** is corrupted with gaps. In this situation, the first step in reconstructing the corrupted trajectory is to define a *k*×3*m*-matrix ***N***, that contains only those rows in ***M*** with complete marker information (*k* < *n*). Performing a PCA on ***N*** gives a basis of PC-vectors, ***PC***, that quantify all intercorrelations between the marker coordinates in ***N***. In the second step, another PCA is conducted on a copy, ***N***_*zeros*_, of ***N*** where the columns that had gaps in ***M*** were replaced by zeros, yielding the basis ***PC***_*zeros*_. Third, a transition matrix ***T*** between the two PC-bases ***T*** is determined. Finally, a matrix ***M***_***zeros***_ is defined by replacing the gaps in ***M*** by zeros. Now an estimate for the missing marker’s coordinates can be obtained by calculating a reconstruction matrix ***R*** through the following set of bases transformations [4]:

$$R=M_{zeros}\;PC_{zeros}T\;{\left(PC\right)}^{-1}$$ (1)

This approach is only successful because human motion data is highly intercorrelated, so that the data is sparsely represented in the ***PC***- and ***PC***_*zeros*_-spaces. Hence, only the lower-order PC-vectors contain information about the volunteer’s or patient’s posture while high-order PC-vectors contain mostly noise. Limiting the number of PC-vectors included in the analysis therefore improves the reconstruction accuracy [4]. However, it has been shown that higher-order PC-vectors may also contain valuable information [13]. Previous PCA-based reconstruction algorithms used a fixed number of PC-vectors [4]. In the current paper, we propose to select the number of PC-vectors included in the analysis by setting a threshold ϴ_λ_ on the cumulative sum of normalized singular values. Compared to using a fixed number of PC-vectors, this has the advantage of being data-driven.

A second measure to minimize the error when projecting frames with incomplete information onto the ***PC***-basis is to assign weight-factors to those marker trajectories in ***M*** that are in spatial proximity to the marker whose gaps are to be filled [4]. The current study implemented an automated, data-driven procedure for determining these weight-factors: For a given marker with gaps, the Euclidean distance *d*_*j*_(*t*) to any other marker *j* was calculated for all time-frames that include the trajectories of both markers. Weight-factors *w*_*j*_ were then obtained through a Gaussian function *w*_*j*_ *=* exp(-*d*_*j*_^*2*^*/2*σ^*2*^). Here *d*_*j*_ is the time average of the Euclidean distance and σ is a scaling parameter determining the behavior of the weighting procedure. The weight factor assigned to the available data of the missing marker was denoted *w*_*mm*_, and was treated as a separate parameter in the weighting procedure.

### Reconstruction strategies for gaps in multiple markers

In principle, the algorithm outlined in the previous section can be directly applied to datasets with more than one corrupted marker trajectory. This can be achieved by simply replacing all gapped marker trajectories with zeros when defining ***N***_*zeros*_. However, how to define suitable weight-factors remains unsolved. Omitting the weighing procedure would severely diminish the reconstruction result, but valorizing the neighbors of several markers would cause the reconstruction accuracy to depend on the distribution of the gaps, and would increase the likelihood of artifacts.

An alternative approach is to reconstruct gaps in different markers individually. For successful reconstruction of individual markers one must remove the gaps in other markers from ***M***. This can be achieved by either omitting gap-contaminated marker trajectories, or gap-contaminated time frames. However, both procedures entail loss of information, and finding a suitable tradeoff is not trivial. Further, it is likely that the optimal tradeoff depends on the individual dataset that is analyzed. To meet the challenges listed above, we developed and tested two gap filling strategies. Both algorithms presented and evaluated in the current study are provided in the supplementary materials of this paper.

#### Reconstruction strategy R1: simultaneous reconstruction of multiple gaps

All corrupted trajectories were reconstructed in a single application of eq 1. This was achieved by defining the matrix ***N*** to contain only time frames without gaps in any marker. Further, ***N***_*zeros*_ was identical to ***N***, but all marker-trajectories that had gaps were zeroed. The weight-factors *w*_*i*,*j*_ *=* exp(-*d*_*i*,*j*_^*2*^*/2*σ^*2*^) for each marker *j* with a complete trajectory were determined by using the largest weight calculated for all markers *i* with gaps (here *d*_*i*,*j*_ represents the average Euclidean distance between markers *i* and *j*). Strategy R1 has the two weaknesses that were already mentioned above: First, all weight factors *w*_*i*,*j*_ are only appropriate for filling the gaps in the closest marker, yet they also influence the reconstruction of all other gaps. Second, the number of time frames that define ***N*** is limited to the frames with complete marker data. Valuable information that would be available from incomplete time frames is neglected.

#### Reconstruction strategy R2: consecutive reconstruction of multiple gaps

As stated above, consecutive reconstruction of gaps in multiple markers requires omission of either whole time frames where other markers have gaps, or of whole marker trajectories corrupted with additional gaps. Hence, a decision criterion was needed to determine which of the two procedures was to be taken for any corrupted marker trajectory *h* when reconstructing the trajectory of marker *i*.

How much information one marker can contribute in the reconstruction of another marker depends on how close the markers are in the kinematic chain. The average Euclidean distances were therefore used as a decision criterion for whether marker trajectory *h* with gaps should be omitted or included in the reconstruction of marker *i*. Only markers closer to marker *i* than a given threshold were included in the analysis. This threshold was expressed relative to the average distance *D*_*i*_ from marker *i* to all other markers. We defined a parameter ϴ_D_ that was used as a cutoff criterion: Any marker *h* with gaps and with a Euclidean distance greater than ϴ_D_ ∙*D*_*i*_ was omitted from the analysis. Otherwise, its trajectory was included, but then the time frames in which that marker had gaps were removed from the analysis. If gaps in any of the included markers overlapped in time with the marker being reconstructed, then these markers were omitted from the analysis regardless of the decision criterion.

### Datasets and performance

**Fig 1:**
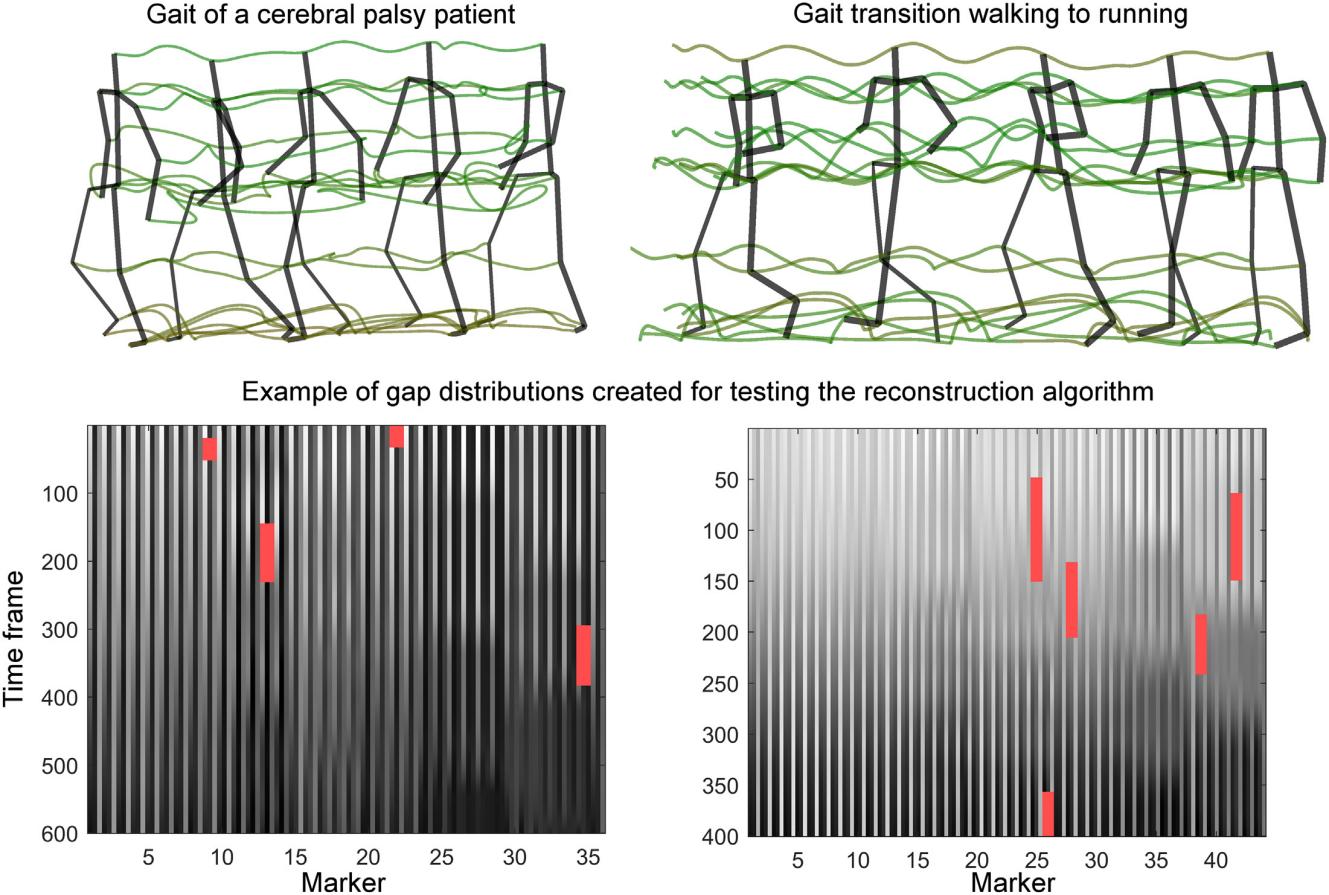
Movement patterns and gap distributions in CP-gait and WalkRun. Top row: Illustration of the movement patterns. Note that certain maker trajectories have been merged to reduce the number of markers. Bottom row: Examples of the measurement matrices of the two movement patterns after being corrupted with gaps. Left matrix: CP-gait, right matrix: WalkRun.

We used three different datasets to investigate the proposed algorithms’ performance in a wide range of applications. The first dataset consisted of the movement pattern of a healthy male subject walking 20 steps on a treadmill. This dataset was used in a proof-of-principle analysis in [4], and was shown to be well-suited for the proposed reconstruction strategies. It contained 37 gap-free, unfiltered marker trajectories, captured at a frame rate of 240 Hz (4300 time frames). The dataset is denoted WalkL in this study. The second dataset consisted of a few steps of a child with Cerebral Palsy. Specifically, the dataset (denoted CP-gait) contained 600 time frames of 35 gap-free, unfiltered marker trajectories captured at a frame rate of 200 Hz. Collection of these data was approved by the regional ethics committee in Norway (REC Central), and the volunteers and their parents gave written informed consent. The third dataset, denoted WalkRun, was downloaded from the HDM05 database (http://resources.mpi-inf.mpg.de/HDM05), and included the transition from walking to running. Specifically, we used the file “HDM_bd_01–03_01_120”, and extracted the time frames 1951–2350. These timeframes contained two steps of walking, before transitioning into running and then capturing three full running steps. The frame rate of these measurements was 120 Hz. A visual representation of the last two datasets is given in the top row of Fig 1. Due to the impaired motor control resulting from Cerebral Palsy, and the sudden change in correlation structure seen during the walk-run transition, we suspected that our reconstruction algorithm, which is based on correlations between markers, might be less successful for these datasets than for the WalkL dataset.

#### Performance testing

To investigate the performance of the proposed algorithms on these datasets, we conducted two different analyses: First, we created gaps in single markers at selected time points. Second, we created gaps in multiple markers at random time points. For the single missing marker case, we created in the CP-gait and WalkRun datasets gaps in the left knee (LKNE), the left shoulder (LSHO), and the left anterior wrist markers (LWRA). The choice of these three specific markers was made because they were believed to reflect markers that were both less suited (LWRA), suited (LKNE) and well suited (LSHO) for this type of reconstruction [4]. We created gaps in either the last half of the time frames, or in 100 time frames in the middle part of the measurements. In the WalkRun dataset, this corresponded to reconstructing a running gait using only walking gait as input, and to reconstruct a gap in the transition phase using both walking and running gaits as input. In the WalkL dataset, we created a gap in the last 3870 time frames of markers LHEE, LANK, LKNE, LASIS, LSHO, LELB, LWRA and LFHD, and used the first 430 frames as input. For this dataset we calculated not only the mean Euclidean difference, but also the mean absolute difference in the three spatial directions. This allowed a direct comparison with the results in [4].

In the second analysis, we created randomly distributed gaps in all three datasets described above. The gaps were random in both anatomical and temporal positions. For the WalkL dataset, 5–15 gaps of duration 20–480 frames (0.08–2 seconds) were created, and then reconstructed using both the R1 and the R2 algorithms. For the shorter CP-gait and WalkRun transition measurements, 2–6 gaps of 20–120 frames (0.1–0.6 s or 0.17–1 s, respectively) were created and reconstructed using R1 and R2. Fig 1 (bottom row) shows examples of corrupted datasets obtained during this procedure. For comparison we also reconstructed the corrupted trajectories using spline interpolation and the DynaMMo algorithm (using 10 hidden dimensions) proposed by Li et al. [7]. The DynaMMo algorithm has been used as a reference to compare gap-filling strategies in other studies [5, 10]. This procedure was repeated 50 times for each dataset, giving a total of 150 reconstructions for each of the two tested algorithms. In all analyses, the performance of the algorithms was quantified by the average Euclidean distance between the reconstructed and the measured marker trajectory.

### Sensitivity analysis

We tested the sensitivity of the R1 and R2 algorithms to changes in the four parameters (*w*_*mm*_, σ, ϴ_λ_, ϴ_D_) by varying these parameters using a one-at-a-time approach. Specifically, we varied *w*_*mm*_ from 10^−5^ to 1 using 15 logarithmically spaced steps, σ from 100 to 1500 [mm] using 15 linearly spaced steps, ϴ_λ_ from 0.86 to 1.0 using 15 linearly spaced steps, and ϴ_D_ from 0 to 2 in 11 linearly spaced steps. The default values for the parameters not being tested were kept constant at *w*_*mm*_ = 0.02, σ = 200 mm ϴ_λ_ = 0.99, and ϴ_D_ = 0.5, which were also the parameter choices used for all other experiments presented in this study. For the sensitivity analysis, we created 20 corrupted copies of each of the datasets, where the gaps were randomly distributed as described in the preceding section. These gaps were then filled using the R2 approach. The parameters’ effect on reconstruction accuracy, quantified by the mean Euclidean distance between measurement and reconstruction, was calculated by subtracting the average reconstruction accuracy of each specific corrupted dataset.

## Results

### Gaps in a single marker

**Fig 2:**
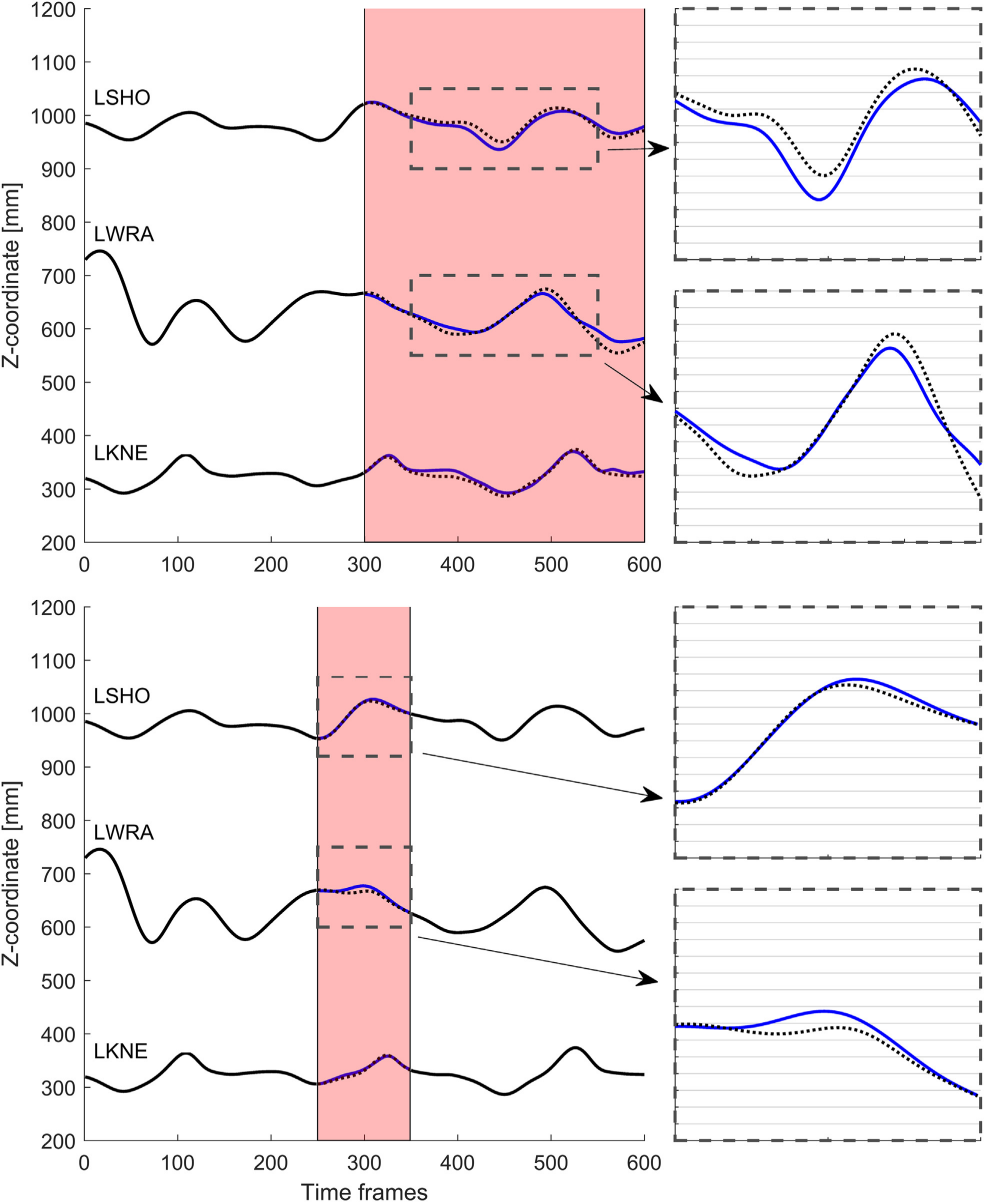
Reconstruction of gaps in single markers in the CP-gait dataset. Shaded red area indicates the gapped time frames. Blue lines represent reconstructed data, black lines input data, dotted black lines reference trajectory. The gridline spacing in the callout-figures is 1 cm.

Table 1 shows results from creating gaps in the markers LWRA, LKNE and LSHO in the CP-gait, WalkRun and WalkL datasets. Gaps in the LWRA marker were less successfully reconstructed than in the LKNE and the LSHO markers. For CP-gait, if we exclude the LWRA-marker, the mean reconstruction accuracy was less than 1.5 cm even if the last half of the trajectory was missing (similar to a marker detachment). However, reconstruction of the LWRA marker was less successful, with an average reconstruction accuracy of 2.8 cm in the same situation. The vertical component of the reconstructed trajectories are plotted in Fig 2.

**Table 1:** Mean Euclidean differences between reconstruction and measurement for gaps in single markers.

Dataset	CP-gait		WalkRun		WalkL
Position of gap	Middle of file (fr. 250–349)	Last half (fr. 301–600)	Transition (fr. 150–249)	Last half (fr. 201–400)	Last 90% (fr 431–4300)
LKNE	6	14	10	25	4
LSHO	8	13	4	15	2
LWRA	18	28	20	36	5

All values are in units of [mm].

**Fig 3:**
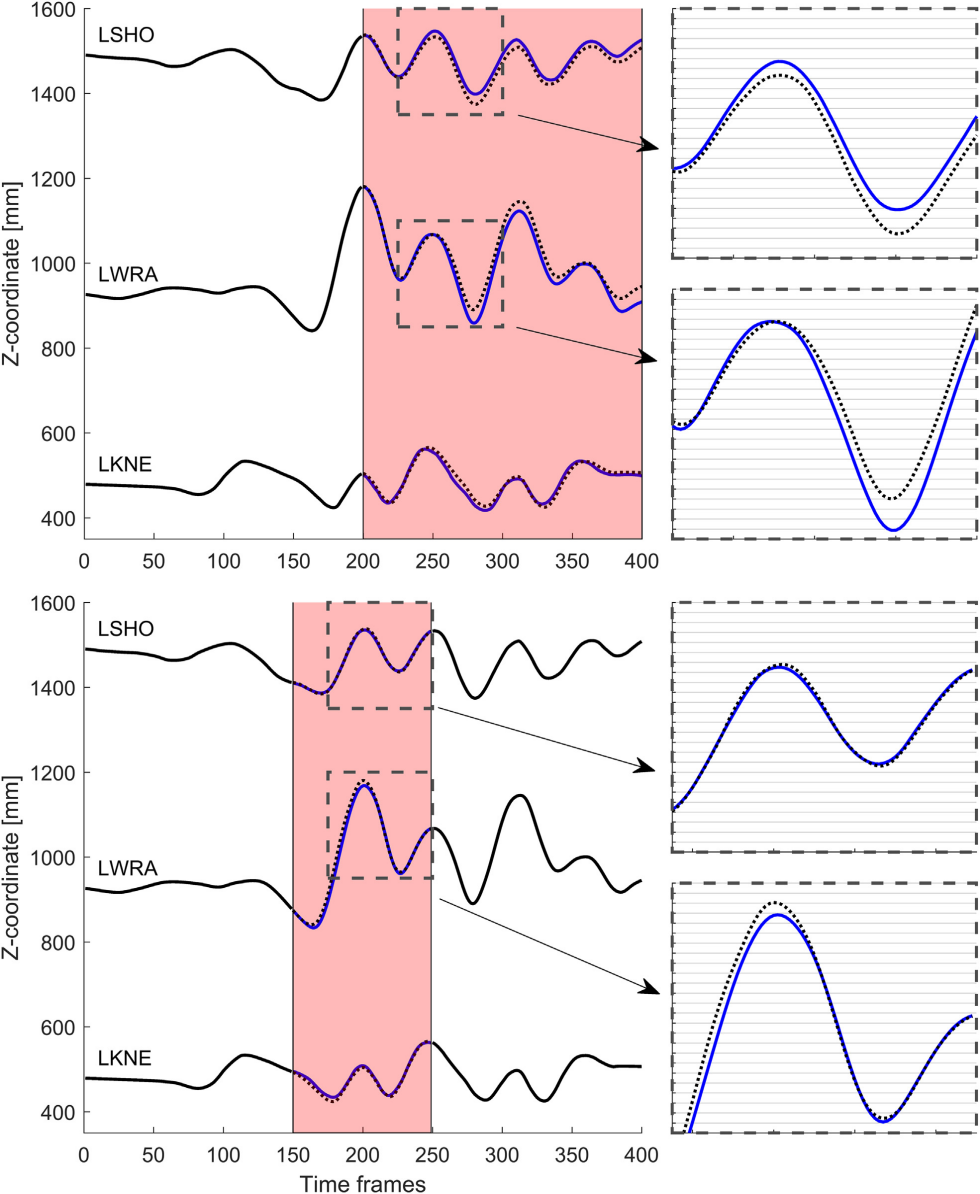
Reconstruction of gaps in single markers in the WalkRun dataset. Shaded red area indicates the gapped time frames. Blue lines represent reconstructed data, black lines input data, dotted black lines reference trajectory. The gridline spacing in the callout-figures is 1 cm.

For the WalkRun dataset, reconstruction of the transition phase gave relatively good results (Table 1). In particular, the LKNE and the LSHO markers both had reconstruction accuracy ≤ 1 cm. However, the error in the LWRA reconstruction was substantially worse (2 cm). Not surprisingly, reconstructing a running gait using only walking as input severely impacted the reconstruction accuracy (Table 1). Fig 3 shows the vertical component of the reconstructed trajectories.

Reconstruction errors in the WalkL-dataset in the three spatial directions are presented in Table 2. Reconstruction of all markers was successful with mean errors < 5 mm in all spatial directions. These results are directly comparable to the results in [4] and indicate an improvement on average by 1.3 mm in each direction (X, Y or Z), corresponding to a relative improvement of 42%.

**Table 2:** Mean differences between reconstruction and measurement for gaps in single markers in the WalkL dataset.

	LHEE	LANK	LKNE	LASIS	LSHO	LELB	LWRA	LFHD
X-dir	3	1	2	3	1	2	2	1
Y-dir	1	1	2	4	1	3	3	1
Z-dir	1	1	2	2	1	1	2	1

X-direction is anterior, Y-direction mediolateral, Z-direction vertical. All values are in units of [mm].

### Gaps in Multiple markers

**Fig 4:**
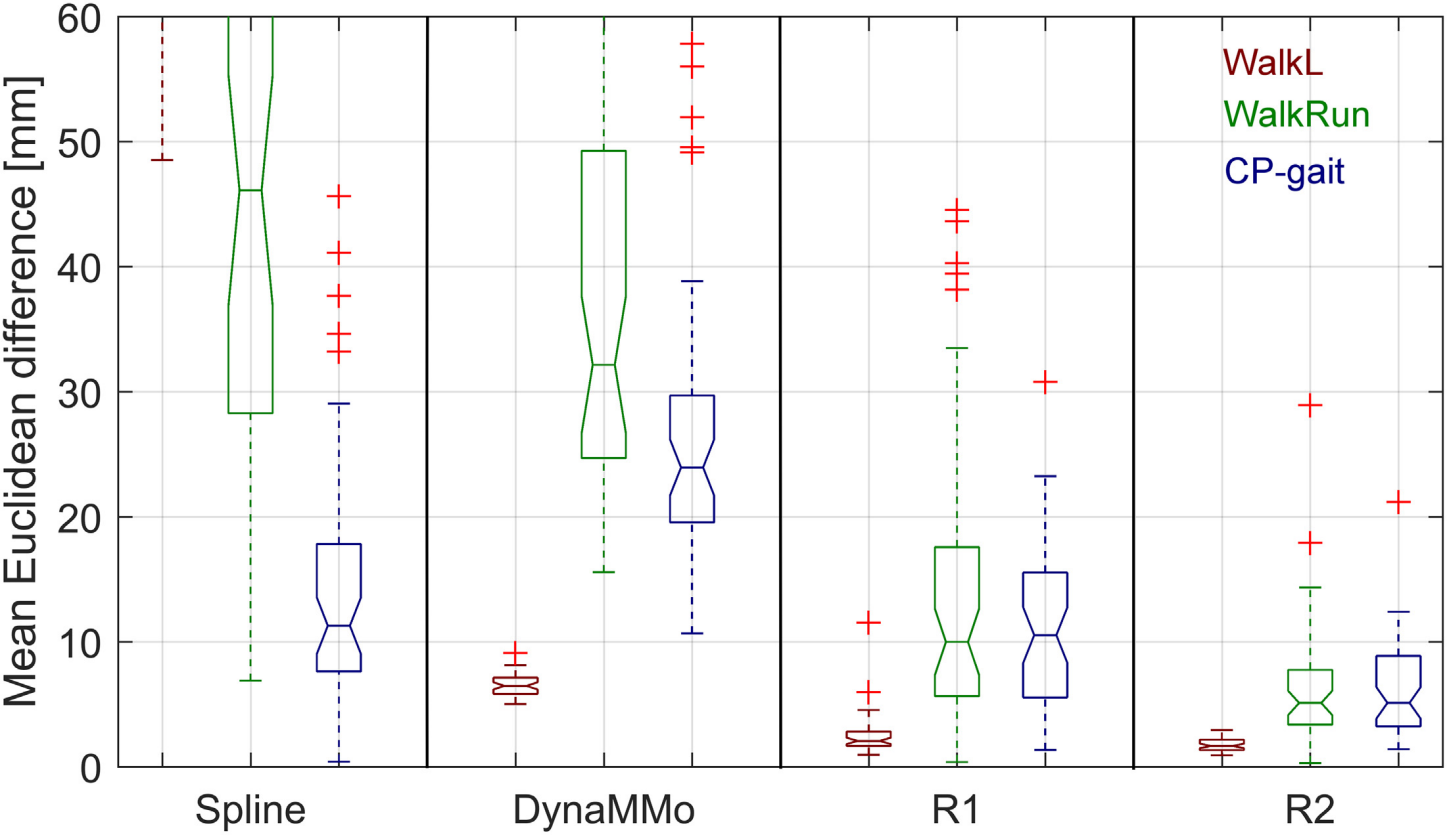
Boxplots of reconstruction accuracies. R1 and R2 are the two algorithms proposed in the current study. Spline interpolation and the DynaMMo algorithm [7] are included for comparison.

**Fig 5:**
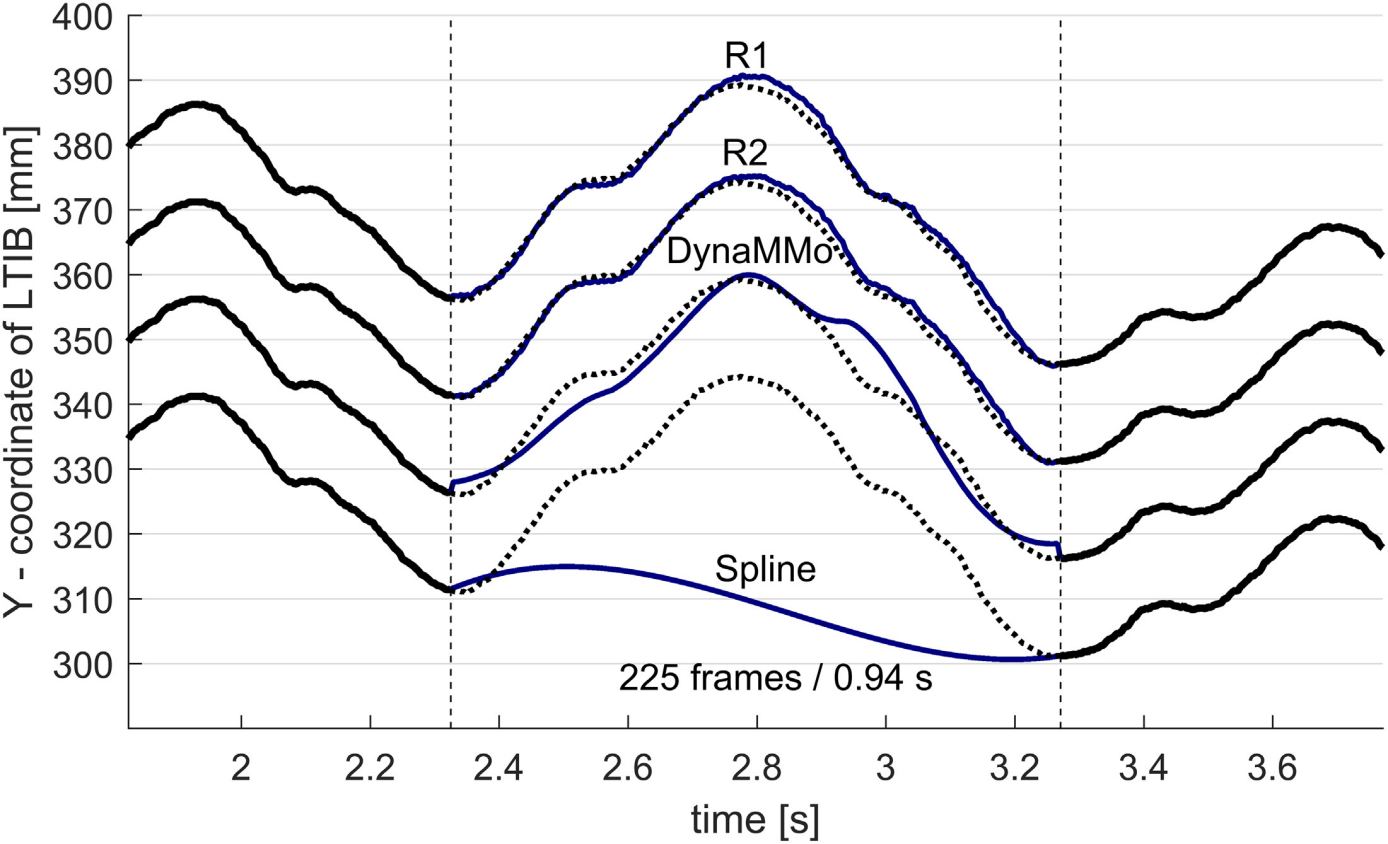
Example reconstruction of a gap in the left tibia marker in the WalkL dataset. The black lines represent the reference trajectories, the blue lines represent reconstructed trajectories for each method. Each reconstruction was shifted by 15 mm vertically for better clarity.

For the WalkL dataset, we obtained high reconstruction accuracy using both the R1 and R2 algorithms. Specifically, the median reconstruction accuracy was 2.1 and 1.7 mm for the R1 and R2 algorithms respectively. The DynaMMo algorithm was also able to successfully reconstruct this dataset, with a median reconstruction accuracy of 6.5 mm. Spline reconstruction was not applicable for filling such highly corrupted datasets, with a median reconstruction accuracy of 116 mm. Fig 4 shows boxplots of the reconstruction accuracy distributions. It shows that for the WalkL dataset, all 50 R2-reconstructions yielded median reconstruction accuracies better than ≤ 3 mm. Fig 5 shows an example of a reconstructed trajectory using the four above mentioned reconstruction approaches.

For the WalkRun dataset, median reconstruction accuracy was 10.0 mm and 5.1 mm for the R1 and R2 algorithms respectively. It is clear from Fig 4 that some reconstructions had substantially worse results, with mean errors up to 29 mm for the R2 algorithm. Both the DynaMMo algorithm and spline interpolation were less successful, with median reconstruction accuracies of 32 mm and 46 mm respectively.

For reconstructions of the CP-gait dataset we found median reconstruction accuracies of 10.5 and 5.1 mm for R1 and R2 respectively. Spline interpolation resulted in a reconstruction accuracy of 12.5 mm, similar to R1, while the DynaMMo algorithm performed slightly worse (median accuracy 24 mm). There were some poor reconstructions of this dataset, even when using the R2 algorithm (mean Euclidean error up to 21 mm, Fig 4).

### Sensitivity analysis

**Fig 6:**
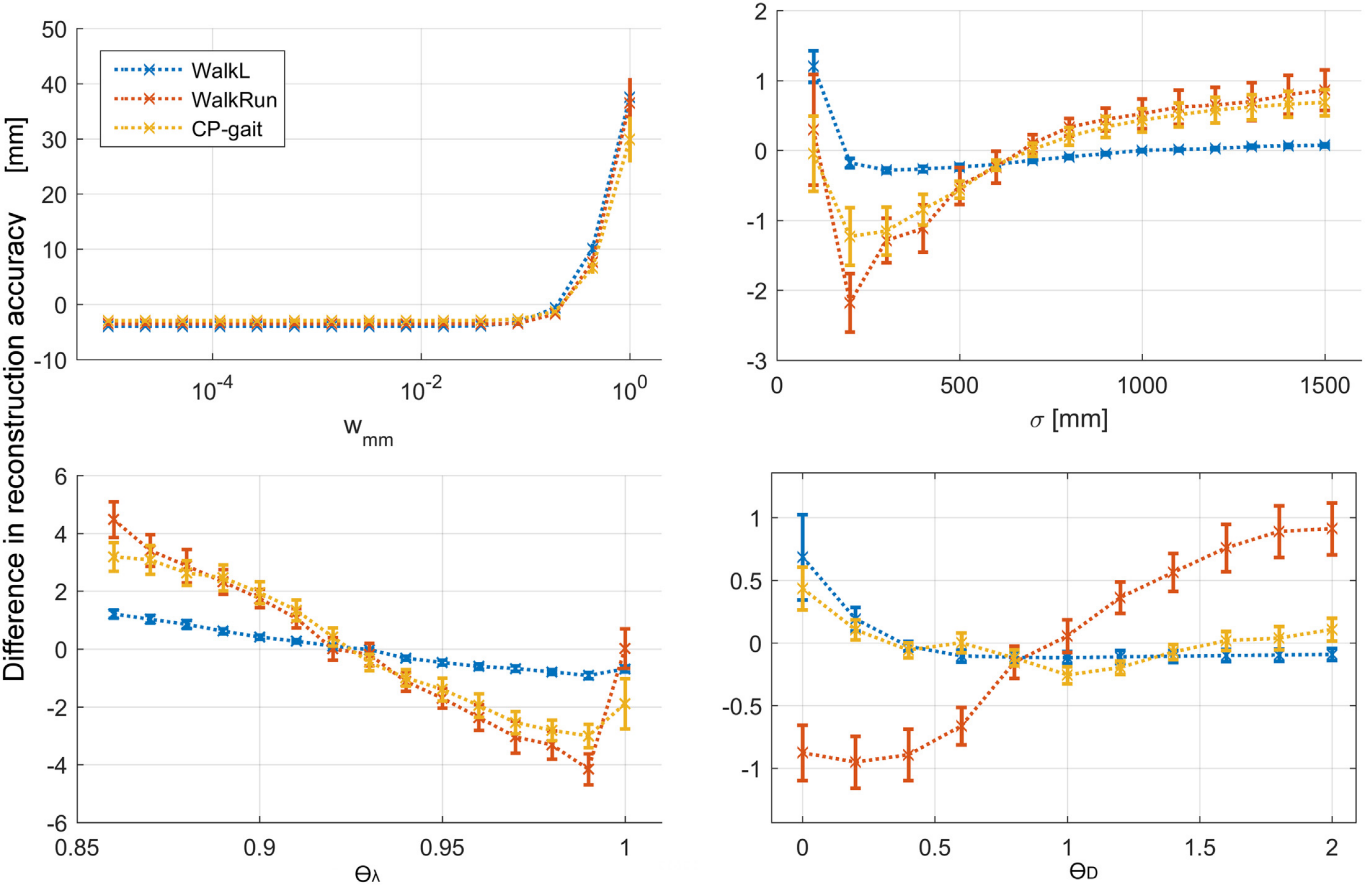
Results of sensitivity analysis. w_mm_: weight factor on missing marker coordinates, σ: scaling of weight factors, ϴ_λ_: threshold on cumulative sum of singular values, ϴ_D_: threshold to discard distal corrupted trajectories. Error bars show the standard error of the mean.

The results of the sensitivity analysis are shown in Fig 6. For the *w*_*mm*_ parameter, choosing a weight substantially smaller than 1 was important for successful reconstruction. The value of *w*_*mm*_ must however be non-zero, otherwise the two PC-bases will be identical and the reconstruction not successful.

For the scaling parameter σ we found a median optimal value of 200 mm, but there was some variation between the datasets: for the highly repetitive WalkL dataset, assigning large weights to many markers did not deteriorate the results considerably. This was not the case for the less predictable movement types, which benefitted from valorizing only the markers relatively close to the marker being reconstructed.

For the cutoff ϴ_λ_ with respect to the normalized variance included in the analysis (i.e. number of PC-vectors considered), we found that the best results were obtained with a value of 0.99 for all datasets.

Lastly, the effect of changing the cutoff threshold ϴ_D_ for discarding or considering corrupted marker trajectories in the analysis differed between the datasets. For the WalkL dataset and the CP-gait dataset, the best results were obtained with ϴ_D_ ≈ 1, while for the very short WalkRun dataset, a substantially smaller parameter value (ϴ_D_ = 0.2) gave the best results. This suggests that in a dataset with very few time frames, favoring time frames at the expense of excluding markers is a good strategy.

## Discussion

The current study accomplished three tasks: First, the gap-filling approach suggested in [4] was improved by automating, in a data-driven way, the selection of several parameters, specifically, the weighing of neighboring markers and the number of PC-components to include. Thereby the reconstruction accuracy was improved on average by 1.3 mm corresponding to a relative improvement of 42% compared to the results in [4]. Second, the current study developed 2 strategies, R1 and R2, for solving the multiple-missing marker problem. Testing of these two algorithms versus both conventional spline interpolation and another openly available gap filling algorithm [7] suggested that the proposed algorithm outperforms both of these approaches. Third, the current study evaluated the performance of R1 and R2 on two datasets (CP-gait, walk-run transition) that were expected to be more challenging for this covariance-based reconstruction approach than the repeated gait cycles (WalkL) tested in [4]. In all three datasets the median R2 reconstruction accuracies for cases with multiple gaps were better than 6 mm. This accuracy is close to typical optical motion capture measurement accuracy [14], and smaller than soft tissue artifacts reported in the literature [15, 16]. However, in the WalkRun and CP-gait datasets R2 also produced in a number of cases reconstruction results that exceeded 10 mm (up to 2.9 cm average error in the worst case). Hence, as always, care must be taken during post-processing and gap-filling results need to be evaluated for plausibility and carefully examined for potential artifacts.

From the experiments with gaps in a single marker at pre-determined time points we found, not surprisingly, that reconstruction of one type of gait using another type of gait as input was not successful. In particular, reconstruction of running gait using walking gait as input resulted in mean Euclidean errors up to 36 mm (Table 1). Also, reconstruction of markers at the distal end of extremities (i.e. LWRA), showed relatively large errors. However, reconstructing markers with ample neighboring markers (LKNE and LSHO) was more successful, with errors ≤ 10 mm for a gap in the walk-run transition and the mid-section of the CP-gait (Table 1).

### Parameter choices

The parameter choices had moderate to high impact on the reconstruction results in the current study. Most important was that the weight factor assigned to the missing markers was small compared to weights put on the neighboring trajectories. The results also indicated that adjusting the parameters to the specific dataset (i.e. to the movement that is reconstructed) can improve the result. Specifically, the less regular CP-gait dataset was most accurately reconstructed when valorizing only the closest markers.

The choice of including or excluding corrupted markers in the reconstruction was affected by the total number of time frames. A reasonable rule of thumb seems to be that datasets with few available time frames will benefit from including many or all of the time frames in the reconstruction. However, if a large number of time frames are available, then including marker trajectories rather than time frames can be beneficial. What constitutes “a few” or “a large number of” time frames is an open question that likely depends on the specific type of movement and the ratio between number of time frames and dimensions of the dataset (i.e. number of markers).

### Comparison to other gap-filling approaches

Our algorithm does not a priori enforce musculoskeletal constraints. Other approaches [17, 18] have been suggested that take such constraints into account, however, these methods require additional input or calibration routines. Our algorithm is conceptually close to the method proposed by Liu and McMillan [8] which uses training data and a random forest classifier to project corrupted data frames onto the PC-space. In the current study the reconstruction is accomplished through a series of coordinate transforms in combination with a weighing algorithm that modulates the covariance structure in the data by valorizing those markers which likely hold information about the missing marker position. Thus we can avoid the use of recursive procedures or the need for training data. In principle our algorithm can also be used with training datasets and we would speculate that training data might improve reconstruction accuracy, particularly for short datasets. However, training data is not always available. A gap-filling algorithm for which training data is not necessary may find a wider range of application areas.

### Limitations

The accuracies reported in the current paper are specific to the datasets tested in the current study. Datasets containing other types of human movement, or containing fewer markers, might be less suitable for the proposed algorithm. In particular, we expect the algorithm to be less suited for non-cyclic movement patterns, such as a squat-jump or a soccer kick. We speculate that successful reconstruction of such datasets is possible by using training data, however, this would require alterations to the current algorithm in addition to further testing. In any case we recommend that researchers evaluate the applicability of the proposed algorithm for their particular datasets, for example by reconstructing artificially created gaps in a trial with complete marker information. For this purpose, we will provide our algorithm for creating gaps in given datasets in the supplementary materials.

## Supporting Information

S1 Matlab Code Zip-file with implementations of the R1 and R2 algorithms in Matlab code. The zip-file contains two files: PredictMissingMarkers.m which contains the reconstruction algorithm, and GapfillGUI.m which provides a graphical user interface to be used with PredictMissingMarkers.m.
(ZIP)

S1 Datasets Zip-file with the three datasets used in this study. The data is provided as matlab (.mat) files, which has an array “Data” holding the measurement matrix.
(ZIP)
